# Mutation accumulation in a hybrid parthenogenetic vertebrate

**DOI:** 10.1093/molbev/msag214

**Published:** 2026-09-03

**Authors:** Zoë L Müller, Joshua Orlando Rivera, Kathleen Currie, Hairo Isaac Rios Carlos, Sean M Rovito, Corey Roelke, Matthew Fujita

**Affiliations:** Department of Biology, Amphibian and Reptile Diversity Research Center, University of Texas at Arlington, Arlington, TX, USA; Department of Biology, Amphibian and Reptile Diversity Research Center, University of Texas at Arlington, Arlington, TX, USA; Department of Biology, Amphibian and Reptile Diversity Research Center, University of Texas at Arlington, Arlington, TX, USA; Unidad de Genómica Avanzada, Centro de Investigación y de Estudios Avanzados del Instituto Politécnico Nacional, Irapuato, Guanajuato, Mexico; Unidad de Genómica Avanzada, Centro de Investigación y de Estudios Avanzados del Instituto Politécnico Nacional, Irapuato, Guanajuato, Mexico; Department of Biology, Amphibian and Reptile Diversity Research Center, University of Texas at Arlington, Arlington, TX, USA; Department of Biology, Amphibian and Reptile Diversity Research Center, University of Texas at Arlington, Arlington, TX, USA

**Keywords:** molecular evolution, mutations, sex, parthenogenesis

## Abstract

Asexual lineages are thought to experience elevated extinction rates compared with sexual species, yet direct evidence for the underlying genetic causes remains scarce. Muller's ratchet predicts that the absence of recombination in asexual organisms facilitates the accumulation of deleterious mutations, thereby reducing long-term fitness. Here, we test this hypothesis in the hybrid-origin, parthenogenetic whiptail lizard *Aspidoscelis tesselatus* by integrating short-read RNAseq and long-read IsoSeq data from both the asexual lineage and its parental sexual species. We reconstructed phased transcripts for *A. tesselatus* to quantify mutation accumulation relative to the parental sexual species. Comparative analyses revealed elevated ω ratios in both parental genomic complements (subgenomes) of the parthenogenetic lineage, consistent with accelerated accumulation of nonsynonymous mutations. Structural variant analyses identified multiple indels in expressed transcripts predicted to disrupt protein domains. Functional annotation indicated that genes affected by both single-nucleotide variants and indels were enriched for roles in chromatin organization, apoptosis regulation, and transcriptional control. While both parental subgenomes showed similar evolutionary patterns, the maternal complement exhibited more structural and missense mutations than the paternal complement. Together, these results provide evidence that mutations accumulate in asexual *A. tesselatus* in genes involved in core cellular functions, supporting theoretical predictions that Muller's ratchet contributes to mutation accumulation in asexual lineages.

## Introduction

Within vertebrates, asexual lineages are rare, and known examples appear to be of recent origin (Kearney et al. 2009; Neaves and Baumann 2011; Fujita et al. 2020; Barley et al. 2022; Freitas et al. 2022). Despite the lower energetic costs and short-term demographic advantages of asexual reproduction (i.e. the cost of males), sexual reproduction remains the predominant form of reproduction in vertebrates (Smith 1971). An important hypothesis explaining why clonal reproduction is less common than sexual reproduction in vertebrates is Muller's ratchet, which posits that deleterious mutations accumulate throughout the genome in asexual populations due to the absence of recombination and segregation (Muller 1964). In asexual populations, whole genomes act as a single, linked unit in the absence of recombination, reducing the effective population size and thereby diminishing the role of purifying selection in removing deleterious mutations (Hadany and Feldman 2005). In addition to the accumulation of deleterious mutations, asexual populations likely have difficulty with adaptation; without recombination, beneficial mutations are unable to quickly come together as they can in sexual populations, a hypothesis known as the Fisher–Muller effect (Fisher 1930; Muller 1932; Crow and Kimura 1965). Unable to escape decreasing fitness and with an inability to quickly adapt, asexual populations eventually face extinction (Lynch et al. 1993; Muller 1964). Meanwhile, with sexual reproduction, recombination allows for the regeneration of the least-loaded genotype classes (genotypes with the fewest deleterious mutations), providing opportunities for selection to eliminate deleterious mutations and thereby avoiding the effects of Muller's ratchet (Hill and Robertson 1966; Haigh 1978). Thus, asexual reproduction has notable disadvantages that can lead to extinction, whereas sexual reproduction largely avoids those processes that can lead to deleterious mutation accumulation.

Some of the best models of genome evolution in the absence of sex are sex chromosomes, including neo-sex chromosomes. Heteromorphic sex chromosomes (Y or W) have drastically reduced recombination, resulting in genomic decay and mutation accumulation due to Muller's ratchet (Bachtrog 2013). For example, in male *Drosophila miranda*, the accumulated transposable elements on the Y chromosome are incompletely silenced, leading to a surge of repeat expression, which contributes to faster aging (Wei et al. 2020). The few studies that have examined nucleotide-resolution mutation accumulation across the whole genomes used ω ratios (*dN/dS*) between asexual and sexual populations to measure the accumulation of nonsynonymous substitutions (Jaron et al. 2021). This value measures the rate of nonsynonymous mutation accumulation versus the rate of synonymous mutation accumulation, with values of one and higher indicating positive selection, and values lower than one indicating purifying selection (Nei and Gojobori 1986). In asexual lineages, the accumulation of nonsynonymous mutations when compared to sexual relatives can be interpreted as the relaxation of purifying selection when the ω ratios approach 1 (Boussau et al. 2011; Pellino et al. 2013; Maldonado et al. 2022). For example, asexual populations of New Zealand mud snail (*Potamopyrgus antipodarum*) had higher ω ratios in their mitochondrial genomes than sexual populations of the same species (Neiman et al. 2010). Additionally, asexual *Timema* stick insects had higher ω ratios in one mitochondrial and two nuclear loci when compared to sexual species in the same genus (Henry et al. 2012). While smaller effective population sizes or lowered selective pressures may have contributed to elevated ω ratios in these asexual lineages, the authors note that the most likely explanation for these results is that deleterious mutation accumulation resulted from less effective purifying selection in the asexual species. However, some studies have found no evidence of mutation accumulation in asexual lineages compared to sexual relatives. For example, ancient asexual oribatid mite species show no evidence of mutation accumulation, perhaps because of large population sizes that allow for the maintenance of purifying selection (Brandt et al. 2017). In many asexual populations, the irreversible loss of the least-loaded class of individuals leads to a progressive increase in mutation load (Haigh 1978). With the lack of recombination, genomes are effectively linked, and the least-loaded class cannot be regenerated once lost. As deleterious mutations accumulate, variance in fitness among individuals increases, and fewer individuals contribute to future generations, thus reducing the effective population size (Haigh 1978; Charlesworth et al. 1994). Thus, the large effective population sizes of oribatid mites may counteract this mechanism (Brandt et al. 2017). A lack of mutation accumulation has also been observed in the asexual genomes of *Cobitis taenia* (spined loach), *Warramaba virgo* (grasshopper), and several hexapod species (Brandt et al. 2019; Kočí et al. 2020; Kearney et al. 2022).

Among vertebrates, only squamates (lizards and snakes) have multiple lineages that reproduce by obligate parthenogenesis, in which individuals reproduce clonally without any contributions from males (Fujita et al. 2020). Obligate parthenogenesis has arisen independently in nine families of squamates but represents less than 1% of squamate diversity (Fujita et al. 2020). In parthenogenetic species, homologous chromosomes are duplicated, with crossing over occurring between duplicated copies of identical chromosomes (Neaves and Baumann 2011). Though these species arise from hybridization, this form of recombination between identical sister chromosomes still exposes the species to mutation accumulation (Neaves and Baumann 2011). Despite these expectations, asexual vertebrate mutation accumulation has not been well studied, with a few exceptions. One notable example is in the mitochondrial genome of asexual *Aspidoscelis* lizards, where 6 out of 13 protein-coding genes exhibited ω ratios that were twice as large as those found in the sexual species, supporting the accumulation of nonsynonymous mutations in parthenogenetic lineages (Maldonado et al. 2022). Additionally, previous work identified large tandem duplications in the mtDNA of *Aspidoscelis* lizards resulting from hairpin structures at duplication endpoints (Stanton et al. 1994). In the asexual gecko lineage *Heteronotia binoei*, large tandem duplications appear in the mitochondrial genome, likely the result of slipped-strand mispairing (Fujita et al. 2007).

To better understand Muller's ratchet and its impacts on protein-coding gene evolution, it is vital that mutations are identified and annotated to understand their impacts on parthenogenetic lineages. In this study, we use one parthenogenetic vertebrate lineage, *Aspidoscelis tesselatus*, to investigate mutation accumulation compared to sexual species. We hypothesize that mutations accumulate in the parthenogenetic lineage because of the relaxation of purifying selection. We expect that mutation accumulation will negatively impact protein function in the asexual lineage by disrupting open reading frames and affecting key catalytic or structural sites. As mutations accumulate throughout the protein-coding genome of asexual lineages, we expect functional genes to be disrupted. To investigate this question, we study multiple types of mutations, including single-nucleotide polymorphisms (SNPs) and indels, identified by phasing *A. tesselatus* subgenomes and comparing them with each respective parental species, *Aspidoscelis marmoratus* and *Aspidoscelis septemvittatus*. To understand how mutation accumulation impacts the genome and lineage fitness, we conduct functional annotation and mutation effect analyses. This study enhances our understanding of how asexual reproduction influences the accumulation of mutations in a vertebrate parthenogenetic lineage.

## Results

### Transcriptome assembly and IsoSeq data filtering

**Figure 1:**
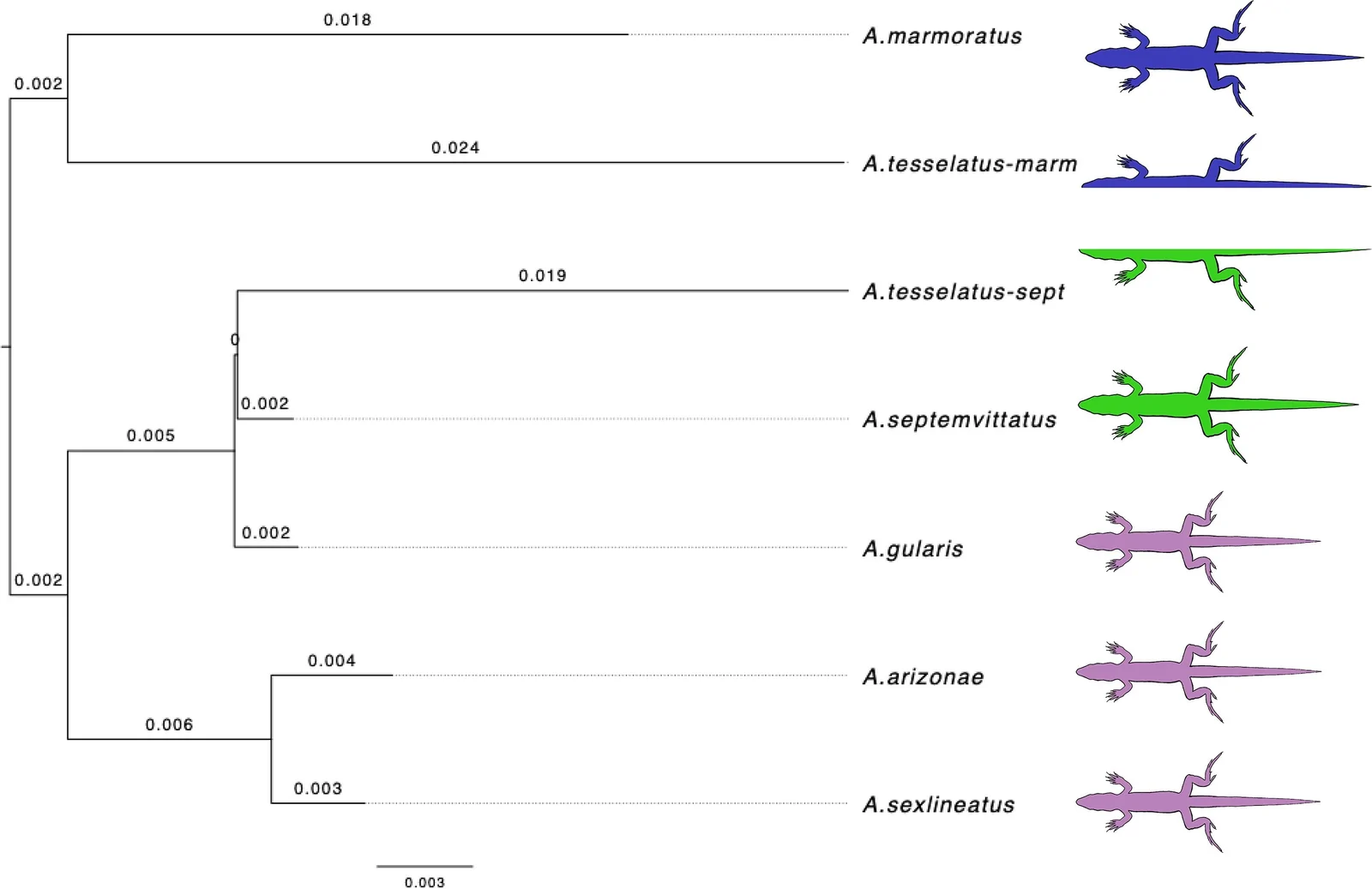
Phylogenetic relationships between parental species, subgenomes of *A. tesselatus*, and nonparental sexual species. This maximum-likelihood phylogeny was inferred with IQTREE2 using the GTR + FO substitution model with the concatenated orthologous sequences. Branch lengths represent substitutions per site. Colors denote lineage identity: blue = *A. marmoratus* lineage, green =*A. septemvittatus* lineage, and purple =outgroups.

We sequenced one transcriptome with Illumina short reads for each nonparental sexual species (*Aspidoscelis gularis*, *Aspidoscelis arizonae*, and *Aspidoscelis sexlineatus*) and five transcriptomes for the parthenogenetic *A. tesselatus* (Fig. 1). Illumina produced, on average, 93.88 million 150 bp reads per sample (Table S1; Table S2). After filtering and clustering transcripts, the total number of transcripts ranged from 21,772 to 64,064, with coverage ranging from 47.57× to 95.78× per gene (Table S1). The percent completeness from BUSCO averaged 54.34%, with a minimum of 34.2% to a maximum of 66.9% completeness (Table S2). Because the transcriptomes are from one tissue type, low BUSCO completeness percentages are expected and consistent with previous single-tissue transcriptome studies (Thorstensen et al. 2023). The average length of transcripts from CD-HIT is consistent across samples (668 bp).

We obtained IsoSeq data for three individual lizards: one *A. marmoratus*, one *A. septemvittatus*, and one *A. tesselatus.* An average of 302,135 consensus reads were generated per lizard, circularizing ∼76% of the total reads (Table S3). *Isoseq cluster* generated approximately 225,339 clusters per individual, which were then collapsed into roughly 10,701 nonredundant, unique isoforms with *isoseq collapse* (Töpfer and Tseng 2018). We used the *isoseq collapse* output to run BUSCO to estimate the completeness of the sequence data. The BUSCO scores with the *tetrapoda* database ranged from 15.9% to 44.7%, with *A. tesselatus* yielding the lowest score and *A. septemvittatus* having the highest (Table S2). The *A. tesselatus* IsoSeq dataset was excluded from the study due to its low BUSCO score.

### Transcriptome phasing

After running OrthoFinder for each *A. tesselatus* sample individually, we identified an average of 195 homeologs per sample (sequences in a single species that originated from different ancestral species), for a total of 972 orthologs. From these orthologs, we phased approximately 78%, or 766 1:1 orthologs (sequences across all species that evolved from a common ancestral gene), with the parental haplotypes with *homologizer*. We filtered out sequences with poor alignments, which left 579 orthologs for the mutation accumulation analysis. Ortholog recovery was largely sample specific; no orthologs were shared across all five individuals in either subgenome. However, we found 128 of 579 orthologs recovered in more than one individual. Each ortholog was maintained independently to preserve any variation between individual sequences (Boussau et al. 2011; Maldonado et al. 2022).

Using the raw-read phasing approach, in which reads were phased by mapping them to each parental Isoseq transcriptome, we phased an average of 2,201,595 variants per individual within the *A. marmoratus* subgenome (39% of variants) and 2,330,894 variants within the *A. septemvittatus* subgenome (46% of variants) (Table S4). We removed an average of 712,781 variants for low mapping quality (MAPQ) or high mismatch scores, accounting for ∼14% of variants (Table S4).

With the phased datasets, we estimated nucleotide diversity between *A. tesselatus* individuals. We found a median of *π* = 0.00122 for the *A. marmoratus*-derived subgenome and a median of *π* = 0.00239 for the *A. septemvittatus*-derived subgenome. Both values are consistent with previously recorded nucleotide diversity estimates in *A. tesselatus* (0.0017) (Maldonado et al. 2022). These low levels of diversity among individuals are consistent with a single, shared origin of the lineage and support treating the sampled individuals as replicates in subsequent analyses (Taylor et al. 2003; Maldonado et al. 2022).

### Mutation accumulation analysis

**Figure 2:**
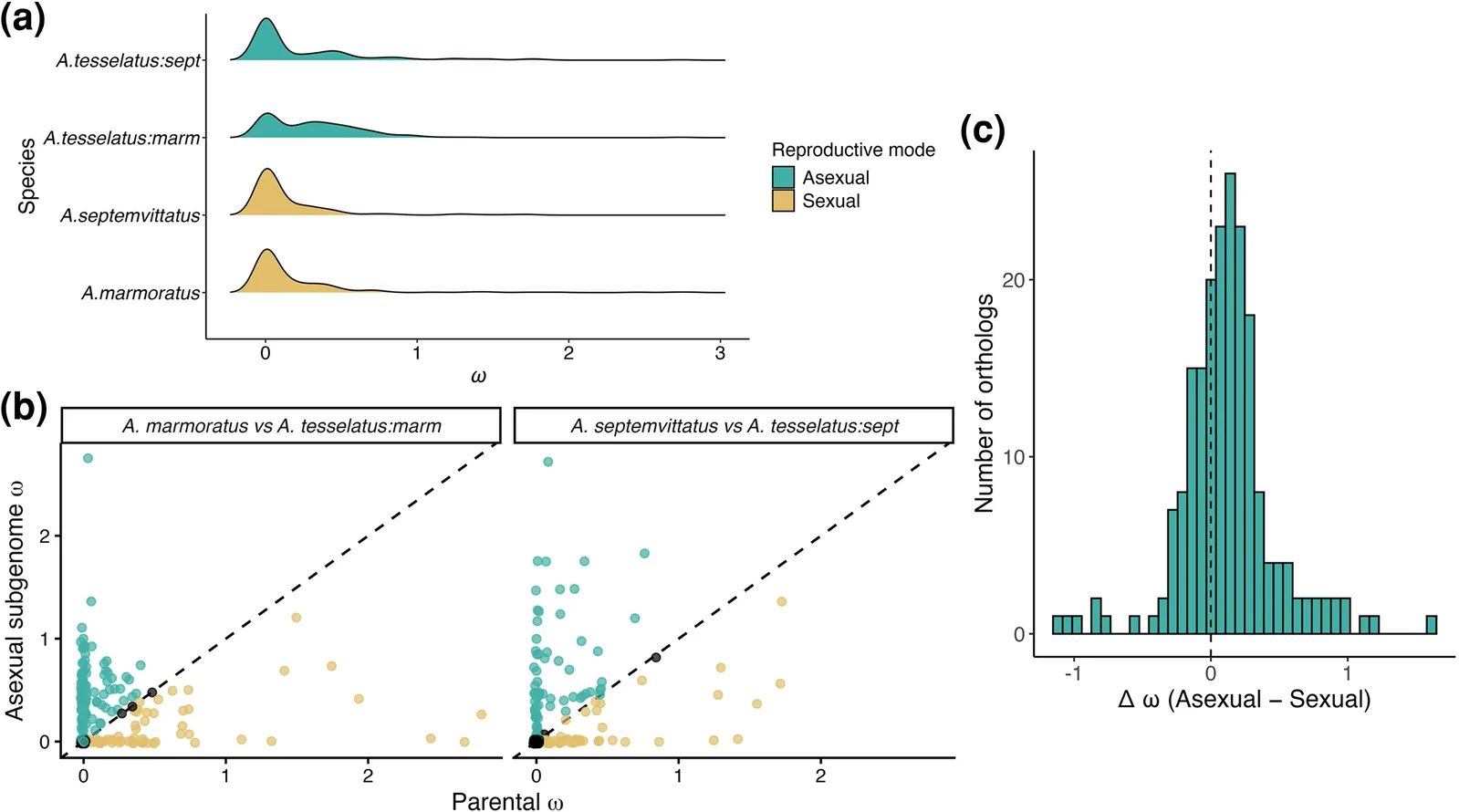
Elevated ω in parthenogenetic *A. tesselatus* subgenomes relative to sexual progenitor species. We removed orthologs with ω greater than 3 for any *codeml* model for visualization. a) Distribution of ω ratios from the four-branch *codeml* output for each parental and parthenogenetic phased branch. Density ridges show the distribution of per-ortholog ω values. The yellow represents the sexual branches, while the teal denotes the asexual phased branches. Mean ω values are higher in both *A. tesselatus* subgenomes (*A. tesselatus-marm* =0.318, *A. tesselatus-sept* =0.263) than in the parental sexual lineages (*A. marmoratus* =0.198, *A. septemvittatus* =0.151). *N* = 204 orthologs. b) Pairwise comparison of ω ratios between each *A. tesselatus* subgenome and its corresponding sexual progenitor species. Each point represents a single ortholog (*N* = 204). The dashed line indicates equality of ω ratios between the asexual subgenomes and the sexual parent. Orthologs above the dashed line have ω values that are higher in the asexual subgenomes than in the sexual progenitor species (green), whereas orthologs with ω values higher in the sexual progenitor are below the line (yellow). Orthologs where ω values are equal between the subgenomes and the parents are on the line, colored black. A total of 127 out of 204 orthologs exceed this threshold in the *tesselatus-marm* comparison and 76 out of 204 in the *tesselatus-sept* comparison. c) Per-gene differences in ω ratios between reproductive modes. The dashed line indicates Δ ω = 0. Bins to the left of the dashed line represent genes, where the ω ratio was higher in the parent progenitor, whereas bins to the right of the dashed line represent genes, where ω was higher in the asexual subgenomes. *N* = 204 orthologs.

We ran *codeml* for 579 phased orthologs, both independently and in a concatenated dataset, using three separate models. We found the asexual versus sexual model (two-ratio branch model) fit the data better than the single ω ratio for 94 orthologs, indicating significant variation in the sexual and asexual branch ω values in those genes based on likelihood ratio tests (LRT) (median LRT = 6.33, range = 3.85–122.11, df = 1, all *P* ≤ 0.0497) (Fig. 2). In eight orthologs, the four-ratio branch model better represented the data than the two-ratio branch model, where each branch has a significantly different ω value (median LRT = 8.66, range = 7.22–83.41, df = 2, all *P* ≤ 0.027) (Fig. 2). For most genes, the ω values were not significantly different between the asexual and sexual branches (Fig. 2). In 84 of the 102 significant orthologs, however, the ω was higher in the asexual branches (median ω = 0.501) than in the sexual branches (median ω = 0.0412), providing evidence for mutation accumulation in the parthenogenetic lineage (Wilcoxon rank-sum test, *W* = 37,063, *P* = 2.65e−31) (Fig. 2). With the alignments concatenated, the two-ratio model fit better than the single *ω* ratio model, but the four-ratio model was not a significantly better fit (Table S5). In the concatenated dataset, the *ω* was significantly higher in the asexual branches (ω = 0.526) than in the sexual branches (ω = 0.303) (*P* = 6.589e−29). This higher estimate relative to the per-gene median likely reflects the influence of longer or more substitution-rich genes in the concatenated alignment, which contribute disproportionately to the parameter estimate compared to the equal weighting of genes in the median calculation.

To test whether the observed mutation accumulation resulted from a single, shared selective regime between the asexual and parental species, we ran PAML's *evolver* function to simulate 700 codon datasets using the phylogenetic tree generated with IQTREE2. Across the distribution of simulated genes, we did not observe any significant difference in the asexual and sexual ω ratios between the two-ratio and four-ratio models (Table S6). Because we found no variation between the sexual and asexual branches in the simulated data, our observed findings deviate from the null hypothesis that a shared selective regime led to the observed variance in ω values.

We extended our simulations to test if variation in effective population size (N_e_) could produce the mutation accumulation observed in the empirical data. We designed two simulations in SLiM5 to generate three populations: two diverged parental lineages and one asexual lineage generated from a single hybridization event with the parental populations (Haller et al. 2026). Each simulation output included 700 codon datasets, with sequences from each parental population and each subgenome for the asexual lineage. One simulation ran with a smaller population size in the asexual population, while the other had all population sizes standardized. In the unequal N_e_ dataset, 92 out of 700 simulated genes better fit the two-ratio model than the one-ratio model (median LRT = 84.8, range = 7.31–193, df = 1, all *P* ≤ 0.0258) (Table S7). Of the 92 significant genes, 52 had higher *ω* in the asexual population (asexual median = 0.382; sexual median = 0.178), supporting that reduced N_e_ results in mutation accumulation (Wilcoxon rank-sum test, *W* = 11,488, *P* = 9.59e−16). No orthologs better fit the four-ratio model than the two-ratio model, supporting a single ω value for each reproductive mode in this dataset. In the equal N_e_ dataset, no ortholog fit the two-ratio or four-ratio model better than the one-ratio model, indicating that a universal ω rate best fits this scenario (Table S7). Based on these simulation results, a reduction in the effective population size of the asexual lineage affects mutation accumulation.

**Figure 3:**
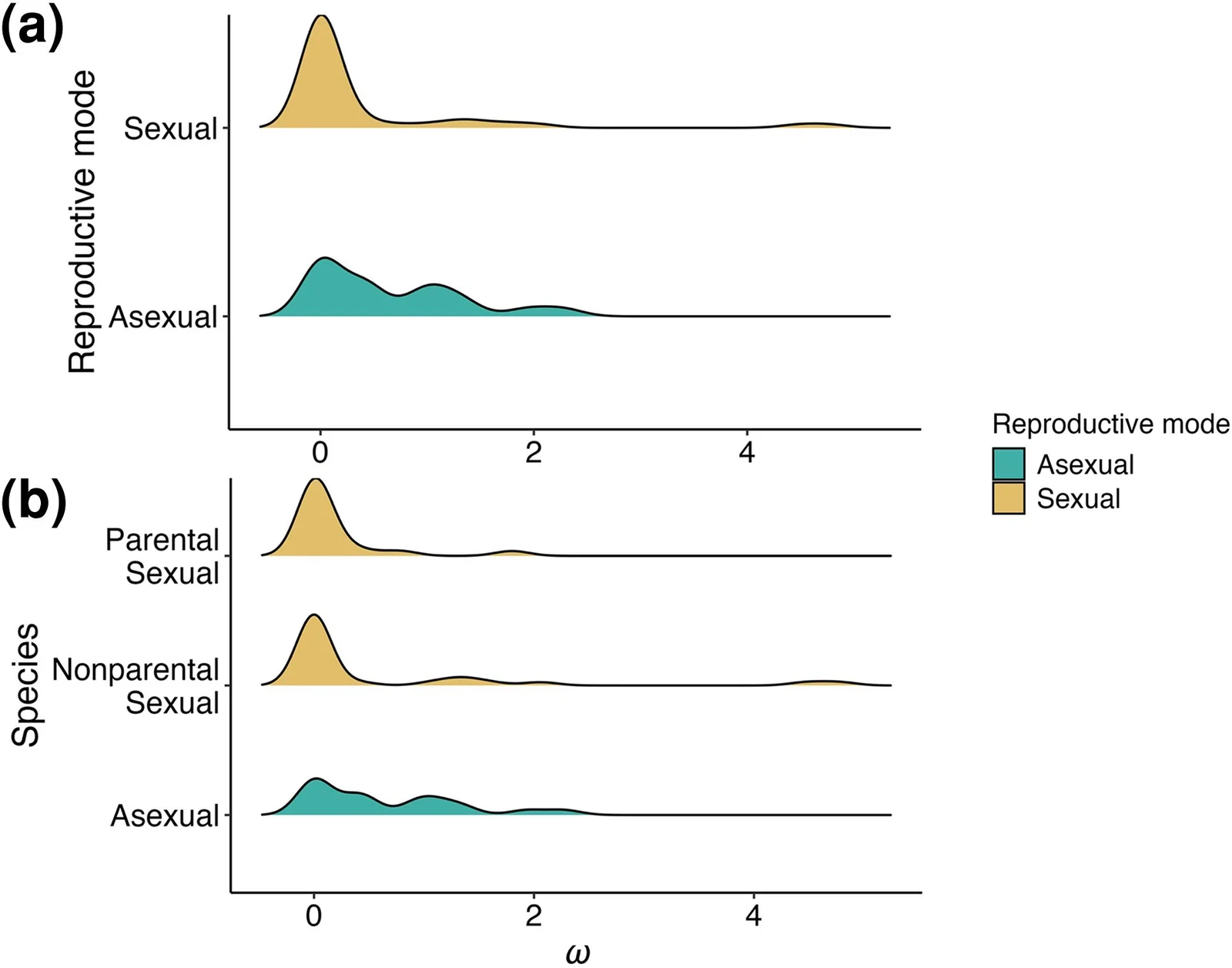
Distribution of ω ratios across reproductive modes and species comparisons support mutation accumulation in *A. tesselatus* subgenomes from *codeml* with the parental, nonparental sexual, and asexual species (*N* = 43). a) Distribution of ω ratios for the seven-ratio model grouped by reproductive mode. b) Distribution of ω ratios for the seven-ratio model grouped by the parental, nonparental, and asexual categories.

We also compared the nonparental sexual species with the parental and parthenogenetic branches to test if mutation accumulation exceeded the background variation in ω in the genus *Aspidoscelis*. We identified 43 single-copy orthologs in all seven species. With likelihood ratio tests, we found the asexual versus sexual model fit the data better than the overall ω model in ten orthologs (median LRT = 6.63, range = 4.09–28.87, df = 1, all *P* ≤ 0.0432) (Fig. 3). Additionally, the nonparental sexual versus parental sexual versus asexual model (three-ratio) was a better fit for the data than the asexual versus sexual model in three orthologs (median LRT = 5.7, range = 4.88–6.04, df = 1, all *P* ≤ 0.0271) (Fig. 3). The model comparing all branches against each other fit the data better than the three-ratio model in only one ortholog (LRT = 12.76, df = 4, *P* = 0.0125) (Fig. 3). Out of these 14 significant orthologs, 12 had higher ω values in the asexual branches (ω = 0.409) than in the sexual branches (ω = 0.0001) (*W* = 250, *P* = 2.015e−8) (Fig. 3). With the concatenated dataset, we found that all the branch ratio models fit better than the single ω model (Table S8). We found that the seven-ratio model, where each branch has its own ω value, best fit the data (Table S8). In that model, the asexual branches had significantly higher ω than both the parental sexual and nonparental sexual species (Table S8).

### Selection analysis

We ran RELAX on 353 ortholog alignments, where ω values from the two-ratio comparison did not exceed three. At the individual ortholog level, however, patterns were heterogeneous. We classified 188 orthologs as under purifying selection (*k* > 1, ω < 1), 126 orthologs as under relaxed selection (*k* < 1), and 4 as under positive selection (*k* > 1, ω > 1) in the asexual branches (Fig. S1a). Genes classified as under positive selection were annotated as involved in mitochondrial respirasome assembly and reproductive processes. Genes under purifying selection were annotated as involved in G2/M transition of the mitotic cell cycle, apoptotic process regulation, DNA replication, and mitotic G2 DNA damage checkpoint signaling. Meanwhile, we found genes under relaxed selection involved in mRNA transport, regulation of DNA-binding transcription factor activity, and regulation of GTPase-mediated signal transduction.

To further evaluate the relationship between selection intensity and substitution rates, we fit linear models testing whether parental ω or the RELAX parameter *k* predicted ω in the asexual subgenomes. Parental ω was not a significant predictor (Fig. S1b; *P* = 0.38), whereas *k* showed strong predictive power (*P* < 2.44e−10). Consistent with this result, we observed a positive correlation between *k* and the shift in ω (ω_asexual_ − ω_sexual_) (Fig. S2; Spearman's ρ = 0.239, *P* = 1.09e−5), which further supports the variation in ω we identified in the *codeml* models. Finally, a Fisher's exact test revealed a significant association between selection regime (intensified vs. relaxed) and elevated ω in the asexual branches (*P* = 0.0173), supporting that genes experiencing relaxed selection are more likely to exhibit increased substitution rates in *A. tesselatus*.

### Indel analysis

**Figure 4:**
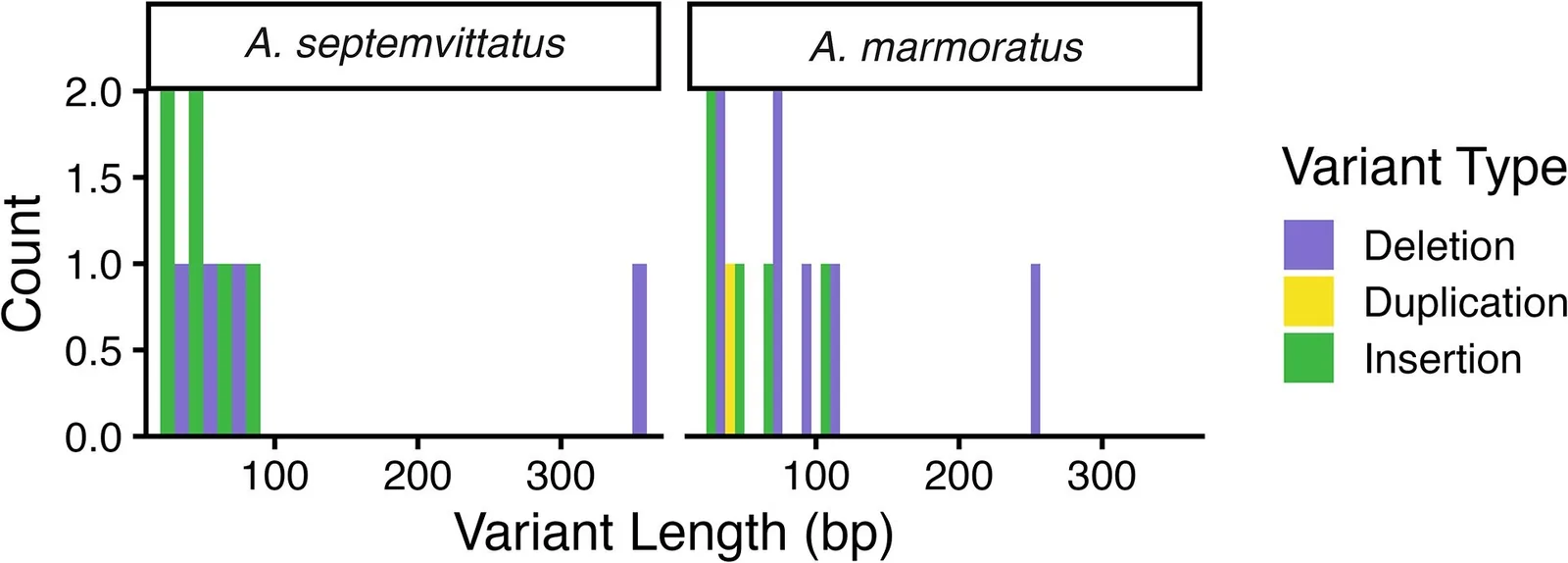
Asymmetric accumulation of indels in the *A. tesselatus* subgenomes. This figure shows a histogram of indels from *A. tesselatus* subgenomes based on the variant length. The bins are 20 bp long.

We used 765 *A. tesselatus* transcripts with start and stop codons in the open reading frame for each sample in this analysis. We removed four variants from the analysis as they represented poor alignments rather than true indels. For the *A. marmoratus* alleles, there were 14 total indels identified, with one inserted duplication, five insertions, and seven deletions based on the comparison with the *A. marmoratus* parental (Fig. 4). These indels ranged in size from 23 to 255 bp (Fig. 4). For the *A. septemvittatus* alleles, we classified ten indels, with six insertions and four deletions (Fig. 4). Most indels were less than 100 bp, but one deletion was 354 bp long (Fig. 4). We also annotated the indels and found that they impacted genes involved in fatty acid catabolic process, monocarboxylic acid catabolic process, and macromolecule localization. Additionally, one of the indels interrupted an RNA-binding motif. From our SUPPA alternative splicing analysis, we found no signatures of alternative splicing in these variants.

### Functional annotation and mutation effect

**Figure 5:**
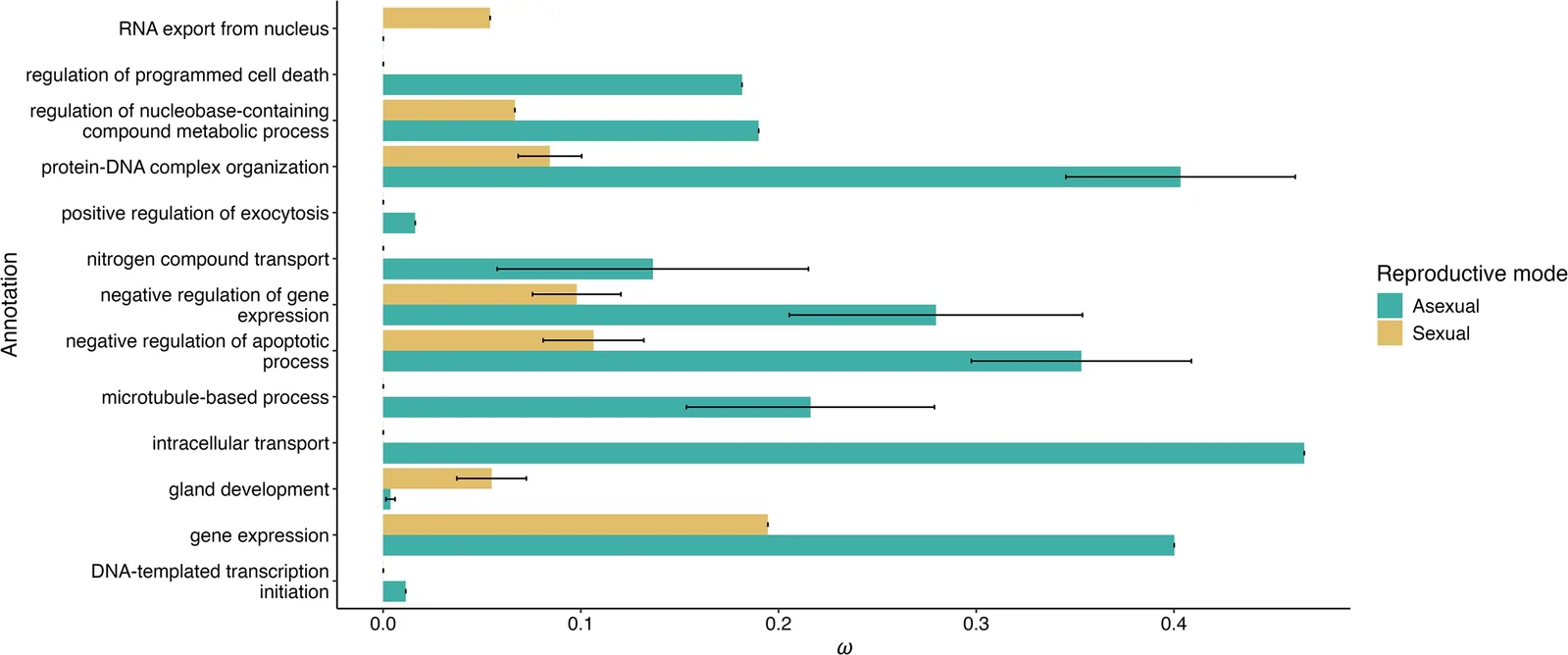
Functional enrichment analysis results with ω ratio comparisons from the two-ratio asexual versus sexual model from *codeml* (*N* = 204).

We annotated 154,447 transcripts from the IsoSeq data, which were then matched to the orthologs used in the mutation accumulation study. We annotated 218 orthologs, which were used for functional enrichment analysis. Database for annotation, visualization, and integrated discovery (DAVID) functional enrichment analysis condensed the annotations into 167 categories, which were further condensed once only orthologs with ω ratios < 3 were retained. We then had 13 categories that were used for analysis. Out of the 13 categories, 11 had significantly higher ω ratios for the asexual branches compared to the sexual species (Fig. 5). These included genes associated with transcription, gene expression, intracellular transport, microtubule-based processes, apoptotic processes, exocytosis, protein–DNA–complex organization, metabolism, and cell death (Fig. 5). In contrast, the sexual branches had higher ω ratios for two annotation categories, including gland development and RNA export from the nucleus (Fig. 5). Overall, there is evidence that nonsynonymous mutations accumulate in protein-coding genes.

**Figure 6:**
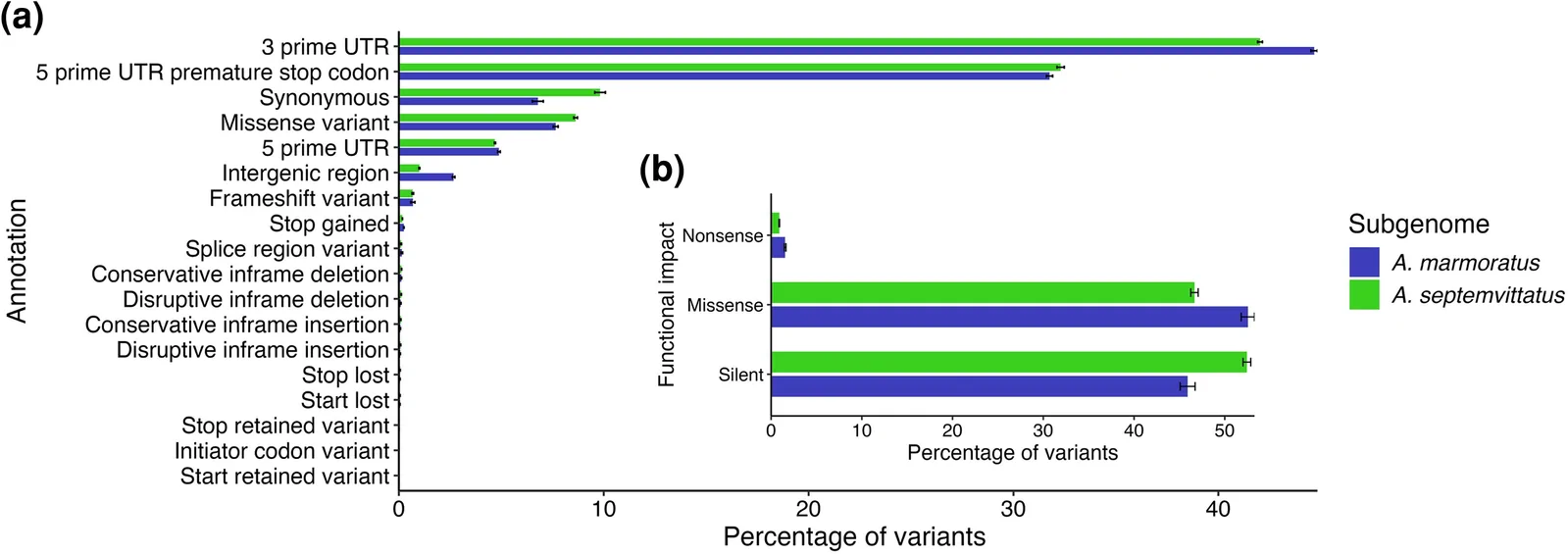
Mutations primarily impact noncoding regions of the *A. tesselatus* transcriptomes. a) Distribution of variant effects across annotation categories identified with SnpEff. Bars represent the percentage of variants assigned to each functional category of the two phased *A. tesselatus* subgenomes (*tesselatus-marm*, blue; *tesselatus-sept*, green). The majority of variants occur in untranslated regions (3′ UTR and 5′ UTR) and synonymous sites. b) Proportion of variants grouped by functional impact class for each subgenome. Values represent the percentage of total variants within each subgenome assigned to each category. Nonsense mutations include stop gained, frameshift, stop lost, and start lost variants. Silent mutations include synonymous, start retained, and stop retained variants. Missense mutations include missense, coding sequence, and inframe deletion and insertion variants. Other variant types in a) are not included in the functional impact classes.

We annotated mutation effects for all individuals for each subgenome with the raw-read phased dataset. Most variants were found in the 3′ untranslated region (UTR) or 5′ UTR regions, potentially impacting protein production and gene expression (Fig. 6a) (Steri et al. 2018). As 3′ UTR regions are characterized by binding sites for miRNAs and ribosome binding proteins, variation in these regions could greatly influence gene expression (Steri et al. 2018). The second most common mutation effect was premature stop codons in the 5′ UTR region, which could impact protein production (Fig. 6a) (Steri et al. 2018). These findings indicate that mutations are accumulating in untranslated regions, suggesting that functional impact may arise primarily through gene regulation rather than changes in coding regions. Additionally, we found further evidence for haplotype divergence between the *A. tesselatus* subgenomes. In all individual *A. tesselatus*, we found a trend where the *A. marmoratus* subgenome had more coding-impact variants (missense) than the *A. septemvittatus* subgenome, which were more enriched for neutral variation (silent variants) (Fig. 6b). We did not find statistical significance between the subgenomes (0.096 *≤*  *P*  *≤* 1), however, the effect sizes of the missense, nonsense, and silent categories were all large (*r* > 0.90), indicating that the variation between subgenome mutation effects is highly consistent across individuals.

## Discussion

This study utilized transcriptomic and IsoSeq data to investigate the genomic consequences of asexuality in the parthenogenetic whiptail lizard, *A. tesselatus*. We find evidence for mutation accumulation in *A. tesselatus* compared to its parental species, *A. marmoratus* and *A. septemvittatus*. While our results are consistent with expectations under Muller's ratchet and therefore provide strong evidence for mutation accumulation in the parthenogens, we cannot fully disentangle its effects from other processes, such as hybrid origin, differences in effective population size, or lineage-specific selective pressures. Importantly, our analyses are based on single-tissue transcriptomes and therefore represent only the expressed portion of the genome in the liver. Our results do not capture genome-wide patterns of mutation accumulation and are biased toward genes expressed in this tissue and under stronger functional constraint.

By phasing the hybrid parthenogenetic transcriptomes into parental subgenomes, we were able to collect valuable information regarding patterns of mutation accumulation in *A. tesselatus*. The *A. tesselatus-marmoratus* subgenome yielded the highest number of deleterious mutations (Fig. 2; Table S5), suggesting a potential bias in mutation accumulation in *A. tesselatus*. We also found a trend of the *A. tesselatus-marmoratus* subgenome accumulating more missense than the *A. tesselatus-septemvittatus* subgenomes across all individuals (Fig. 6). Although we did not find significant variation between the phased subgenomes, the distinctiveness of the *A. tesselatus-marmoratus* subgenome warrants further study of haplotype divergence in parthenogenetic whiptail lizards. These findings could be preliminary evidence of Meselson's effect, which could mitigate the impacts of Muller's ratchet (Welch and Meselson 2000).

Meselson's effect posits that in an asexual lineage, the subgenomes evolve independently due to the absence of recombination (Villegas et al. 2024; Öztoprak et al. 2025). Meselson's effect has been identified as a major factor in avoiding the “dead-end” fate of asexual lineages, as one subgenome may be preserved while the other diverges rapidly, preserving intraindividual genetic diversity (Villegas et al. 2024; Öztoprak et al. 2025). In hybrid-origin asexual lineages, this can be difficult to observe as the subgenomes are diverged at the time of hybridization. However, a recent study found that the Amazon molly (*Poecilia formosa*), a hybrid-origin asexual vertebrate, showed strong evidence for Meselson's effect by comparing both parental and subgenome divergence from a reconstructed ancestral genome (Ricemeyer et al. 2026). Researchers found that asexual subgenomes diverged faster from the reconstructed ancestral genome than the sexual parentals (Ricemeyer et al. 2026). Meselson's effect has great potential to slow the effects of Muller's ratchet, preserving asexual lineages. This pattern should be investigated further in this system using whole genomes.

We found significant variation between the asexual and sexual lineages in our simulated dataset with unequal effective population sizes, suggesting that a smaller N_e_—and greater influence of genetic drift—plays a major role in mutation accumulation in the asexual lineage. *A. tesselatus*, *A. marmoratus*, and *A. septemvittatus* differ in reproductive mode, in geographic range, and potentially in historical demography (Jones and Lovich 2009). These differences likely reflect variation in historical effective population size, which influences the efficacy of selection and the accumulation of mutation. By explicitly testing how variance in effective population size impacts mutation accumulation, we find that reduced effective population size alone is sufficient to produce the elevated ω observed in the asexual lineage, consistent with nearly-neutral theory (Ohta 1973). Notably, the simulated results closely mirror our empirical findings, supporting the hypothesis that neutral processes (e.g. increased influence of genetic drift) drive mutation accumulation (empirical: 84 out of 102 genes with significantly increased ω in the asexual branches; simulated: 52 out of 92 genes with significantly increased ω in the asexual branches).

We also detected mutation accumulation when comparing *A. tesselatus* to the nonparental sexual species (Fig. 3). This finding indicates a consistent elevation in nonsynonymous substitution rates across the sampled protein-coding genes of *A. tesselatus*. Because this increase exceeds the natural range of ω variation observed across the diverse sexual species, the pattern is consistent with reduced efficacy of purifying selection associated with parthenogenesis, rather than lineage-specific rate variation in the parental taxa. This result provides strong support for mutation accumulation in the parthenogenetic lineage.

We conducted a selection test to determine if the elevated ω ratio in the asexual branches was the result of the relaxation of selection or positive selection. We found evidence for both the intensification and relaxation of selection in *A. tesselatus.* With the expected reduced effective population size in the asexual lineage, the intensification of selection in certain gene ontology (GO) categories could be a sign that certain genes are now so vulnerable to further damage that any mutation is lethal. This pattern is consistent with reduced effective population size, as strongly constrained genes may still experience effective purifying selection, despite reduced efficacy in more weakly constrained regions. In contrast, genes under relaxed selection were involved in processes like mRNA transport—functions that typically tolerate greater sequence variation. Mutations in these genes are likely only mildly deleterious; thus, they could easily become fixed in *A. tesselatus*. We also found evidence that elevated ω in the asexual lineages is driven by shifts in selection intensity rather than inheritance of fast-evolving genes from the parental species. Specifically, parental ω values did not predict ω in the asexual branches, but the RELAX parameter *k* was a strong predictor. Overall, these results are consistent with mutation accumulation driven by relaxed purifying selection while also highlighting that strong functional constraints can maintain intense selection in critical gene categories.

We found mutation accumulation in several important protein-coding genes in *A. tesselatus* (Fig. 5). Broadly, genes associated with functions involved in metabolic processes, gene expression, apoptosis, and intracellular transport were most impacted by mutation accumulation in *A*. *tesselatus* (Fig. 5). The two annotations that had the highest ω ratios in the parental species were gland development and RNA export from the nucleus. As the parental species are sexual, their gland development may be under diversifying sexual selection to improve fecundity.

The indel analysis identified most indels as short deletions and insertions (Fig. 4). However, we identified one inserted duplication in an *A. tesselatus-marmoratus* allele (Fig. 4). When we annotated these variants, we found similar results from our functional annotation analysis, where these indels impacted genes involved in functions like metabolic processes, RNA binding, and macromolecule localization. Because we found no evidence for alternative splicing, these variants represent true indels in the *A. tesselatus* transcriptome. These results provide evidence that mutation accumulation in the parthenogenetic lineage can modify mRNA, impacting protein production. We also find some variation between haplotypes in this analysis, where the *A. tesselatus-marmoratus* branch accumulates more indels than the *A. tesselatus-septemvittatus* branch. Although indel variation is minor, the mutation accumulation and mutation effect results from this study provide preliminary evidence of biased haplotype divergence within *A. tesselatus* subgenomes.

Our results are consistent with those from asexual whiptail lizard mitochondrial genomes, which found that deleterious mutation accumulation doubled in the parthenogenetic lineages (Maldonado et al. 2022). Our indel results are also supported by previous literature, as duplications have been identified in asexual whiptail lizards before, specifically in mtDNA sequences (Moritz and Brown 1987). Similar patterns of mutation accumulation have also been observed in asexual *Timema* insect species and asexual lineages of freshwater snails (Neiman et al. 2010; Henry et al. 2012). There is also evidence for haplotype divergence in asexual mite species, which is predicted to increase evolvability in these lineages (Brandt et al. 2021). With some preliminary evidence of haplotype-specific mutation accumulation, further study should be conducted to investigate divergence in parthenogenetic whiptail subgenomes.

This analysis provides evidence for mutation accumulation in the asexual whiptail lineage, *A. tesselatus*, by classifying both SNPs and indels. With mutation accumulation in the asexual subgenomes, we found evidence for significant relaxation of purifying selection. In contrast, genes under strong purifying selection show evidence of intensified selection in the asexual lineage. This finding contrasts with theoretical expectations of Muller's ratchet, as in the ratchet, the efficacy of selection is reduced, limiting its ability to purge deleterious mutation (Muller 1964; Felsenstein 1974). We found intensified selection in genes involved in processes, such as DNA replication—theory predicts that in asexual lineages, mutation rate modifiers, such as DNA replication genes, may experience stronger purifying selection, as failure of these systems would have disproportionately severe fitness consequences (Gabriel et al. 1993). Gene conversion may contribute to these selection contrasts. In a recent study, researchers found that gene conversion prevented mutation decay in the clonal Amazon molly while still allowing for haplotype divergence (Ricemeyer et al. 2026). Additional analyses should be conducted with whole-genome sequences to identify if gene conversion plays a role in *A. tesselatus*'s contrasting selective landscape. Regardless of the mechanisms allowing for this pattern, these results show an interesting pattern, where vital functions are maintained through extreme selective constraints. Overall, our analysis found evidence that mutations are accumulating in the asexual lineage, *A. tesselatus*. Although intensified selection may help maintain important functions, the parthenogenetic system may eventually exhaust opportunities to compensate for deleterious mutations.

## Materials and methods

### Study species

*A. tesselatus* is an asexual species of whiptail lizard, originating from a single hybridization event approximately 360 Kya between a male *A. septemvittatus* and a female *A. marmoratus* (Barley et al. 2022; Maldonado et al. 2022) (Fig. 1); the sexual parents of the hybrid parthenogen diverged approximately 15 to 25 million years ago (Barley et al. 2022). We also compare substitution rate profiles of *A. tesselatus*, *A. septemvittatus*, and *A. marmoratus* with closely related sexual species, including *A. arizonae*, *A. gularis*, and *A. sexlineatus*, to understand if increased divergence results from mutation accumulation linked to asexuality or background differences in evolutionary rates among lineages (Barley et al. 2022) (Fig. 1).

### Sample collection and sequencing

Lizards were collected in Brewster, Presidio, and Potter counties, Texas, United States, and in Otero County, Colorado. We collected five *A. tesselatus* and one individual for each sexual species (*A. gularis*, *A. marmoratus*, *A. septemvittatus*, *A. sexlineatus*, and *A. arizonae*). After humane euthanasia, the liver tissue was dissected and placed in RNAlater for RNAseq experiments or flash frozen in liquid nitrogen for IsoSeq sequencing (Conroy et al. 2009). We generated both short-read RNAseq and long-read IsoSeq data because they provide complementary information: RNAseq offers high-coverage expression data, while IsoSeq produces full-length transcript sequences that improve gene structure reconstruction and allow more accurate detection of indels in the parthenogenetic transcriptomes. We extracted total RNA from the liver tissue using the Zymo QuickRNA MiniPrep kit (Zymo R1055). We quantified RNA using the Qubit 2.0 fluorometer before preparing cDNA libraries. With the extracted RNA, we removed rRNA using the NEBNext rRNA depletion kit and then created the cDNA libraries with the NEBNext Ultra II Directional cDNA library preparation kit (NEB E7400; NEB E7760). Each sample had unique i5 and i7 indices. Equimolar amounts of each sample were pooled before being sequenced on an Illumina NovaSeq 6000 at the North Texas Genome Center.

For IsoSeq data, RNA was extracted, prepared, and sequenced at Novogene (PacBio Sequel IIe) from one flash-frozen liver from each of three species (*A. tesselatus*, *A. septemvittatus*, and *A. marmoratus*).

### Transcriptome assembly and IsoSeq data preparation

**Figure 7:**
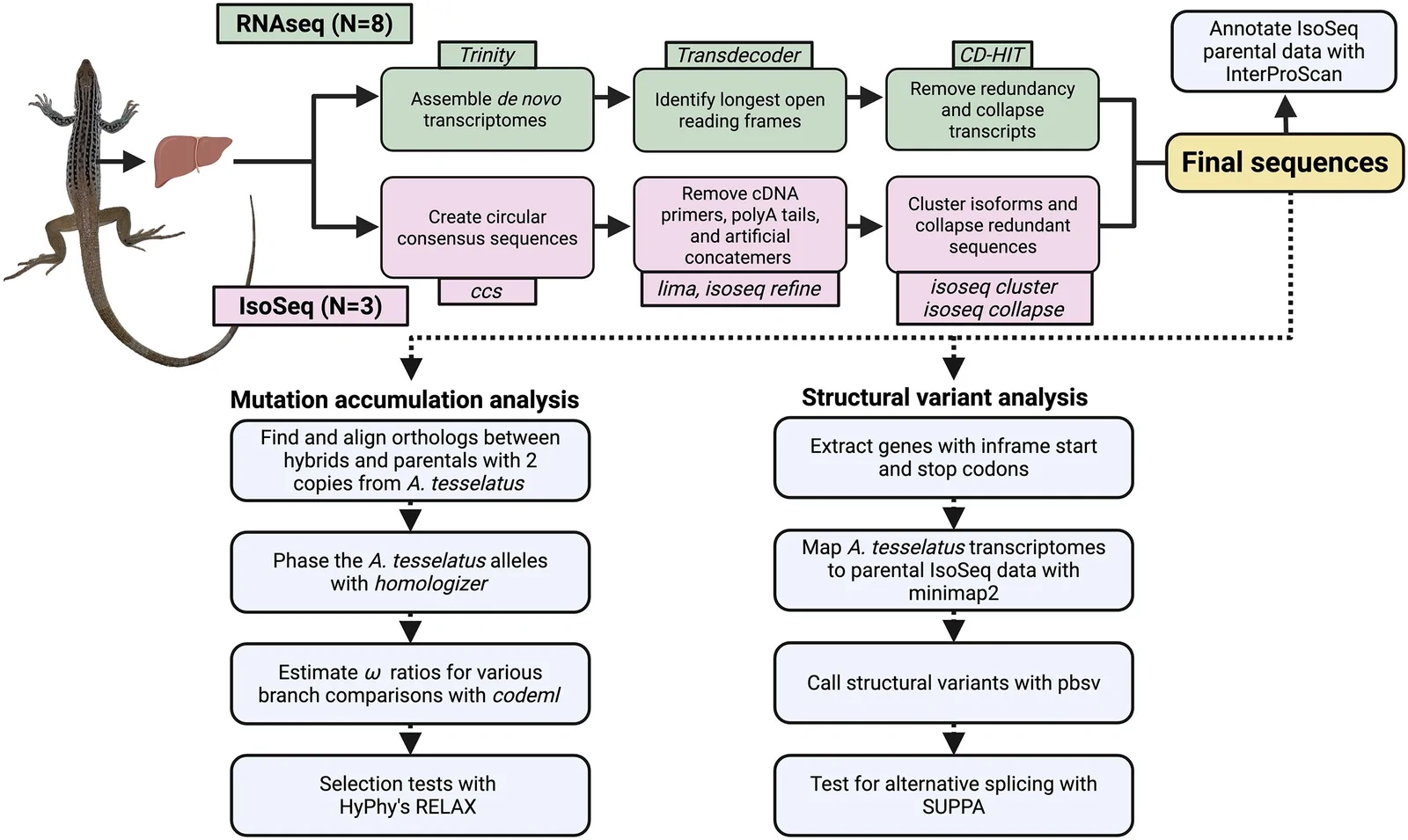
Methodology for IsoSeq filtering, transcriptome assembly, phasing pipeline, and variant calling used for this analysis.

We removed low-quality reads, adaptors, and indices from sequences with Trimmomatic v.0.39 (Bolger et al. 2014). We then assembled RNAseq transcriptomes de novo for all samples using default parameters in Trinity v.2.14.0 (Grabherr et al. 2011). We used TransDecoder v.5.5.0 with default parameters to identify the longest open reading frames of each transcript (Haas 2023). We ran CD-HIT v.4.8.1 with default parameters to remove redundancy and collapse transcripts into putative orthologs (Li et al. 2001) (Fig. 7). We used the CD-HIT output as input to BUSCO v.5.3.2 using the vertebrate gene set to quantify transcriptome completeness (Manni et al. 2021). The filtered transcriptomes from all individuals were used for the following analyses.

For IsoSeq, we generated circular consensus sequences from the subread data using the SMRT Link v.6.0.0 IsoSeq analysis application, following the bulk IsoSeq workflow (Töpfer and Tseng 2018). In the first step, we removed cDNA primers using *lima* and subsequently removed polyA tail and artificial concatemers from the sequence with *isoseq refine* (Töpfer and Tseng 2018). We used *isoseq cluster* to create isoform-level clusters de novo, which were used with BUSCO to determine IsoSeq data completeness (Töpfer and Tseng 2018; Manni et al. 2021). We then used pbmm2 to map the reads to the publicly available *A. marmoratus* genome (GCA_014337955.1) (Ho et al. 2024). This output was used with *isoseq collapse*, which condenses redundant sequences into unique isoforms (Töpfer and Tseng 2018). We used the *isoseq collapse* transcriptome for BUSCO analysis to assess the completeness of the transcriptome (Manni et al. 2021) (Fig. 7). This FASTA output was used for subsequent analyses.

### Transcriptome phasing analysis

To estimate mutation accumulation for parental-specific haplotypes, we phased the *A. tesselatus* transcriptomes with the *A. marmoratus* and *A. septemvittatus* IsoSeq data. We first used OrthoFinder to identify orthologous groups between parentals and *A. tesselatus*, which will serve as the basis for comparison in the mutation accumulation analysis (Emms and Kelly 2019). We distinguish between orthologs (genes diverged by speciation), 1:1 orthologs (single-copy genes present in all species), and homeologs (duplicated gene copies in *A. tesselatus* derived from its hybrid origin). We extracted orthologs with one copy per parental species and two alleles from the *A. tesselatus* transcriptome, and then the *A. tesselatus* alleles were arbitrarily labeled as allele 1 or 2 for the phasing analysis. We aligned these orthologs using Multiple Alignment of Coding SEquences (MACSE) codon-aware parameters and ran Gblocks to refine the alignment by removing hypervariable and gap-rich regions (Castresana 2000; Ranwez et al. 2018). To test if the genes identified were true homeologs (sequences within a species that originated from different parental ancestors), we used IQTREE2 to estimate divergence between the *A. tesselatus* alleles (Minh et al. 2020). If the output tree divided the *A. tesselatus* alleles, assigning one to each parental branch, the homeolog was used for the next analysis. With the finalized set of 1:1 orthologs (sequences across all species that evolved from a common ancestral gene), we used *homologizer* v.1.0.0 to phase the *A. tesselatus* alleles, assigning them to a parental haplotype (Freyman et al. 2023). *Homologizer* uses a tree-based method to infer posterior probabilities of gene copy topology across various loci by switching tip assignments (Freyman et al. 2023) (Fig. 7). The output assigned alleles from *A. tesselatus* to each parental, producing phased copies.

We also phased reads by mapping raw RNAseq from *A. tesselatus* individuals to the parental IsoSeq sequences. We concatenated the parental IsoSeq to phase variants based on mapping quality. We mapped the *A. tesselatus* raw reads to the IsoSeq using minimap2 and then filtered the resulting alignment by removing variants with a quality score less than 30 (Li 2021). After filtering the variants, we used a conservative reciprocal-remapping phasing approach. With this method, we assigned reads to the parental partition only when it mapped with substantially higher confidence (MAPQ expected − MAPQ alternate ≥ 10) and lower mismatch burden to one parental transcriptome than to the alternate parental transcriptome (number of mismatches in the alternate parent mapping − number of mismatched in the expected parent mapping ≥ 2). We classified reads without sufficient discriminatory support as ambiguous and removed them from the analysis. This approach is consistent with practices for allele-specific expression assignment (Krueger and Andrews 2016). The phased datasets were then converted into fastq files with samtools so we could remap them to the single IsoSeq parental sequence (Danecek et al. 2021). We used this phased dataset to calculate nucleotide diversity between *A. tesselatus* individuals and to run SnpEff to examine mutation effects.

To calculate nucleotide diversity (π), we mapped variants to their respective parental IsoSeq transcriptome and then called variants with bcftools mpileup and bcftools call using the haploid model (Danecek et al. 2021). We then used pixy v.1.2.7 to calculate nucleotide diversity between the subgenomes of *A. tesselatus* individuals (Korunes and Samuk 2021).

### Mutation accumulation analysis between *A. tesselatus* and parental species

In this study, we define mutation accumulation as an increase in *dN/dS* (ω), which is the ratio between the rates of nonsynonymous substitutions and synonymous substitutions (Jeffares et al. 2015). With the alignments and tree file from *homologizer*, we ran PAML's *codeml* program to estimate ω (Yang 2007). We used the M0 model to estimate overall ω for each ortholog and then ran *codeml* with the two-ratio branch model to compare sexual to asexual sequences (Álvarez-Carretero et al. 2023). With the branches annotated, this model allows for different ω per branch, and for the two-ratio branch model, we grouped *A. tesselatus-marmoratus* and *A. tesselatus-septemvittatus* into one group to give an overall ω for the asexual subgenomes, and then *A. marmoratus* and *A. septemvittatus* into the second group to estimate ω for the parentals. We also used the branch model for a four-ratio comparison, which estimates ω for each branch, including the parental *A. marmoratus*, the parental *A. septemvittatus*, the *A. tesselatus*-*marmoratus* sequence, and the *A. tesselatus-septemvittatus* sequence. We used likelihood ratio tests to compare the fit of the one-ratio, two-ratio, and four-ratio models with the data. Results from *codeml* for each individual *A. tesselatus* were pooled after analysis for statistical analysis. We retained orthologs identified in more than one individual as separate data points to preserve any interindividual diversity in ω.

### Simulation

To determine if mutation accumulation was the result of neutral evolution, we ran PAML's *evolver* function to simulate a codon dataset. *evolver* simulates nucleotide datasets by modeling neutral evolution with a birth–death process along a user-specific phylogenetic tree (Yang 2007). We generated a consensus tree from all tree outputs from *homologizer* with IQTREE2 and then created simulated codon data using that consensus tree (Minh et al. 2020). We generated 700 simulated codon datasets, each with 600 codons, to better resemble our observed gene dataset. With each simulated codon dataset, we ran *codeml* with the one-ratio, two-ratio, and four-ratio models (Yang 2007). We then compared ω values between the simulated data and the real ortholog data with a *t*-test to determine if the asexual and sexual branches evolving neutrally would have significantly different rates of mutation accumulation. Here, we expect that under neutral evolution, we would see no significant difference between the asexual and sexual branches.

To further investigate neutral evolution in this system, we simulated the effects of effective population size (N_e_) to test whether elevated ω in the asexual lineage could result from reduced N_e_. We used SLiM5 to design a model where nucleotide sequences were randomly generated and allowed to diverge in the two populations for 200,000 years as a burn-in and equilibrium period (Haller et al. 2026). We initiated two mutation types: m1, which was completely neutral, thus representing synonymous mutations, and m2, which had a slightly deleterious effect on the individual, representing nonsynonymous mutations. The nonsynonymous mutations (m2) were assigned fitness effects drawn from a gamma distribution (mean *s* = −0.03, shape parameter = 0.2), whereas the synonymous mutations (m1) had a constant fitness effect of 0. Both mutation types used a dominance coefficient of 0.5. At the end of the burn-in, the parthenogenetic lineage was created by initiating a single hybridization event between one individual from each of two diverged populations. This new individual reproduced solely by cloning, and the subgenomes evolved independently. After 360,000 generations, the simulation was completed by printing out the nucleotide sequences of each parental lineage and the two subgenomes. We ran this simulation for 700 iterations with sequences of 600 codons. To explicitly test the impact of reduced N_e_ in the asexual subgenomes, we ran one set of simulations with equal N_e_ across all populations (150 individuals per population) and another set where the asexual population's N_e_ was a third of the size of the parentals (50 individuals versus 150 for each parental population). With the nucleotide outputs, we ran *codeml* with the one-ratio, two-ratio, and four-ratio models to estimate ω with variable effective population sizes.

### Mutation accumulation analysis with nonparental sexual species

We further examined mutation accumulation in *A. tesselatus* by comparing its subgenomes with those of other whiptail lizard species. This helped us determine whether increased divergence results from mutation accumulation linked to asexuality rather than background differences in evolutionary rates among lineages. In this analysis, we expected that *A. tesselatus* subgenomes would have higher ω values than all the sexual lineages. We ran OrthoFinder with the transcriptomes from the phased *A. tesselatus* sequences from *homologizer*, *A. marmoratus*, *A. septemvittatus*, *A. arizonae*, *A. gularis*, and *A. sexlineatus* (Emms and Kelly 2019). The single-copy ortholog sequences were used for the mutation accumulation analysis. We aligned these with MACSE codon parameters (Ranwez et al. 2018). We used the whiptail lizard tree from Barley et al. (2022) for the *codeml* analysis and placed *A. tesselatus* in the tree by treating each subgenome as sister to the parental species from which it is derived (Barley et al. 2022).

We used the M0 model to estimate overall ω for each ortholog and then ran *codeml* with the branch two-ratio model to compare sexual to asexual sequences (Álvarez-Carretero et al. 2023). To compare asexual, parental, and nonparental sexual species, we used the three-ratio branch model to calculate ω ratios for each group (Álvarez-Carretero et al. 2023). Finally, we compared all branches with a seven-ratio model. We used likelihood ratio tests to compare the fit of the two-ratio, three-ratio, and seven-ratio models with the data (Álvarez-Carretero et al. 2023).

### Selection analysis

To determine if the elevated ω ratios in the *A. tesselatus* subgenomes reflected weakened purifying selection, we ran HyPhy's RELAX function with the parental and asexual subgenome ortholog alignments (Wertheim et al. 2015). RELAX evaluates whether selection is relaxed or intensified along a set of specified test branches (in this case, the asexual subgenomes). If the selection intensity parameter (*k*) is less than 1, there is evidence for relaxed selection in the asexual haplotypes (Wertheim et al. 2015). We ran RELAX for orthologs with ω ratios for the two-ratio model under 3, as extremely high values generally occur when synonymous substitutions are absent, a pattern that typically reflects poor alignments or codon misplacement rather than true biological signal (Álvarez-Carretero et al. 2023).

We ran RELAX using the parental branches as the reference and the two asexual subgenomes as the test branches. We compared gene-wise RELAX estimates of *k* with ω ratios from the *codeml* two-ratio branch model (asexual versus sexual branches) to assess whether changes in selection intensity corresponded to shifts in substitution rates. We also tested whether variation in ω ratios between the parental and asexual branches was associated with RELAX estimates of selection intensity. We grouped our results based on asexual ω ratios and *k* values; orthologs where *k* > 1 and ω > 1 were classified as under positive selection, orthologs where *k* > 1 and ω < 1 were classified as under purifying selection, and orthologs where *k* < 1 were classified as exhibiting relaxed selection (Boussau et al. 2011; Wertheim et al. 2015). We compared the asexual and sexual branches with linear models to test if there is a shift to relaxation in selection in the asexual subgenomes. Based on the expected effects of asexuality on the efficiency of purifying selection, we predicted that the orthologs with low ω ratios in the *A. tesselatus* branches would show little to no relaxation, due to strong functional constraint. In contrast, we expected that orthologs with higher ω values in *A. tesselatus* would show evidence of relaxed selection in the asexual branches, as sequence divergence in these genes is only mildly deleterious.

### Indel analysis

We used complete coding sequences from the *A. tesselatus* transcriptomes (those containing both a start and a stop codon) for the indel analysis. With these filtered sequences, we mapped the *A. tesselatus* transcriptomes to the *A. marmoratus* and *A. septemvittatus* IsoSeq sequences with minimap2, using the output from *isoseq refine* as the IsoSeq reference (Li 2021). To understand the role of indels in parthenogenetic mutation accumulation, we estimated structural variation between *A. tesselatus* and the parental species using *pbsv* (Töpfer and Tseng 2018). *pbsv* is an indel calling and analysis tool designed specifically for IsoSeq reads (Töpfer and Tseng 2018). We ran *pbsv* with relaxed parameters to identify insertions, deletions, and duplications between *A. tesselatus* transcriptomes and the parental IsoSeq data (Heller and Vingron 2021). We then individually inspected each variant with Geneious (v.2025.0.2; Dotmatics) to ensure it was correctly classified. To visualize the output, we used the R package *vcfR* (Knaus and Grünwald 2017). Genes with identified indels were also annotated with BLAST, and these results were used as input to DAVID functional enrichment analysis to generate functional classifications (Camacho et al. 2009; Sherman et al. 2022).

Structural variation may appear in the form of isoforms (e.g. alternative splicing). To test if the variants were the result of alternative splicing, we used SUPPA v.2.4 to detect alternative splicing events in the transcriptomes and IsoSeq data (Trincado et al. 2018). For this analysis, we mapped the sequences to the most closely related species with an annotated genome, *Anolis carolinensis* (GCF_035594765.1), with miniprot (Li 2023). The output gene transfer format (GTF) file was used as the input for SUPPA.

### Functional annotation and mutation effect

We conducted a functional annotation analysis to identify specific functions that might be impacted by increased mutation accumulation. We used InterProScan to annotate the IsoSeq data of one parental species, *A. septemvittatus* (Jones et al. 2014). InterProScan uses various databases to annotate sequences, including Pfam, Reactome, and GO identifications (Griffith et al. 2015). We converted the Reactome annotations to UniProt IDs via the UniProt website (Bateman et al. 2015). We then clustered these annotations with DAVID functional analysis to generate a complete list of functions impacted by mutation accumulation in *A. tesselatus* (Sherman et al. 2022). We used the GO_TERM_BP_FAT annotations for final groupings (Sherman et al. 2022).

For this analysis, we predicted that functional categories involved in sexual traits, such as those important in males (e.g. spermatogenesis) or meiosis-related processes, would show greater mutation accumulation in the parthenogenetic lineage (van der Kooi and Schwander 2014).

To predict the functional effects of mutations, we annotated variants using SnpEff (Cingolani et al. 2012). We called variants from the phased read dataset using bcftools mpileup and bcftools call, with the parental IsoSeq transcriptomes as references (Danecek et al. 2021). Because each subgenome represents a single haplotype, variants were called using the haploid model. We filtered variants to retain sites with a minimum phasing quality of 20 and a base quality of 20. To enable variant annotation, we constructed SnpEff databases for each parental species using the corresponding parental IsoSeq transcriptomes. We generated coding sequences (CDS), protein sequences, and a GFF3 file using TransDecoder, after which the SnpEff databases were built (Haas 2023). We then annotated the filtered phased variants with SnpEff using the respective parental species databases.

## Supplementary Material

msag214_Supplementary_Data

## Data Availability

The scripts used for this study are available in the GitHub repository: https://github.com/zoemullersnakes/MutationAccumulationWhiptails. All transcriptomes, IsoSeq data, simulation files, and intermediate files are available on Dryad. Raw reads are publicly available on NCBI under BioProject PRJNA1381241.
