# Supplementary material for: Mutation accumulation in a hybrid parthenogenetic vertebrate
Source: Mol Biol Evol. 2026 Sep 3;43(9):msag214. doi: 10.1093/molbev/msag214 (PMC13555296; doi:10.1093/molbev/msag214)
Supplement: Supplementary file 1 [file msag214_supplementary_data.pdf]

Supplementary Table 1: Transcriptome processing information.

Sample	Species	Total # cd-hit transcripts	Average length of transcripts (bp)	Coverage	BUSCO % complete	Raw read count
JAM014	tesselatus	59,470.00	669.6	47.57	66.9	76,235,418
KLC066	tesselatus	34,464.00	685.7	89.71	46.8	56,527,052
KLC148	arizonae	55,284.00	664.9	62.54	64.4	100,380,008
KLC157	sexlineatus	45,234.00	725.2	57.6	65.5	72,422,214
KLC167	gularis	48,325.00	671.3	50.27	57.7	107,324,864
KLC168	tesselatus	21,772.00	711.8	95.78	41.4	68,310,836
KLC162	tesselatus	64,064.00	601.9	53.41	57.7	127,107,483
KLC163	tesselatus	40,117.00	617.9	57.06	34.3	142,712,126

Supplementary Table 2: BUSCO completeness information for each transcriptome and IsoSeq transcriptome. The italicized row includes data for a sample we excluded from the study, and the rows shaded grey are IsoSeq transcriptomes.

**Sample**	**Species**	**BUSCO complete single copy**	**BUSCO complete duplicates**	**BUSCO fragmented**	**BUSCO missing**
JAM014	tesselatus	2133	110	8	1103
KLC066	tesselatus	1510	59	684	1101
KLC148	arizonae	2061	99	552	642
KLC157	sexlineatus	2093	105	478	678
KLC167	gularis	1859	75	619	801
KLC168	tesselatus	1338	49	667	1300
KLC162	tesselatus	1860	76	87	1331
KLC163	tesselatus	1100	49	970	1235
JOR1068	*tesselatus*	*557*	436	*68*	*2729*
JOR1048	marmoratus	691	310	100	2253
JOR1112	septemvittatus	852	755	87	1660

Supplementary Table 3: IsoSeq transcriptome processing information. BUSCO % complete is based on the *tetrapoda* database.

**Sample**	**Species**	**CCS read counts**	**Lima counts**	**Cluster count**	**Unique isoform count**	**BUSCO % complete**
JOR1068	tesselatus	95,532	67,821	54,593	3,903	16.6
JOR1048	marmoratus	149,221	122,933	90,173	8,115	29.8
JOR1112	septemvittatus	661,653	599,975	531,253	20,086	47.9

Supplementary Table 4: Phasing output counts for each individual *A. tesselatus* from the raw-read phasing approach.

**Sample**	**No. variants assigned to *A.marmoratus***	**No. variants assigned to *A.septemvittatus***	**No. variants identified as ambiguous**
KLC168	1,228,783	1,089,466	390,997
KLC163	3,705,287	3,538,714	1,032,095
KLC162	3,705,287	3,528,714	1,032,095
KLC066	487,144	1,067,461	250,174
JAM014	1,881,474	2,430,114	858,546

Supplementary Table 5: Summary of *codeml* analyses and comparisons of ω ratios in orthologs in parental sexual species and asexual subgenomes from the concatenated dataset.

Model	ln(L)	Number of free parameters	p-values	ω
One ratio	-189286.715	8	NA	0.47510
Two ratio (sexual vs. asexual)	-189219.990	9	vs. one ratio: 7.2e-31*	Asexual: 0.52640<br>Sexual: 0.30309
Four ratio (individuals)	-189219.594	11	vs. one ratio: 6.6e-29*<br>vs. two ratio: 0.6734039	*A. marmoratus*: 0.3020<br>*A. septemvittatus*: 0.3020<br>*A. tesselatus-marm*: 0.5211<br>*A. tesselatus-sept*: 0.5483

Supplementary Table 6: Summary of *codeml* analyses and comparisons of ω ratios in *evolver* codon datasets.

**Model**	**ln(L)**	**Number of free parameters**	**p-values**	$\boldsymbol{\omega}$
One ratio	-4871.161	8	NA	0.27229
Two ratio (sexual vs. asexual)	-4871.127	9	vs. one ratio: 0.7942713	Asexual: 0.28234<br>Sexual: 0.26699
Four ratio (individuals)	-4871.017	11	vs. one ratio: 0.9622693<br>vs. two ratio: 0.8958341	*A. marmoratus*: 0.27539<br>*A. septemvittatus*: 0.27887<br>*A. tesselatus-marm*: 0.00010<br>*A. tesselatus-sept*: 0.290025

Supplementary Table 7: Summary of *codeml* analyses and comparisons of ω ratios in *SLiM* datasets. These results are from a filtered dataset in which orthologs were excluded if any $\omega > 3$ in the two-ratio or four-ratio models (N = 310). All orthologs were simulated as 1800 bp sequences. For the unequal N_e simulation, the asexual population had 50 individuals, whereas the sexual populations each had 150 individuals. In the equal N_e simulation, all populations had 150 individuals. "Min" represents the minimum value per category, whereas "max" represents the maximum.

**Simulation**	**Unequal N_e (asexual = 50 individuals, sexual = 150)**			**Equal N_e (asexual and sexual = 150)**		
Model	One-ratio	Two-ratio	Four-ratio	One-ratio	Two-ratio	Four-ratio
ln(L) range	*Min* = -2402.135 *Max* = -2290.582	*Min* = -2402.135 *Max* = -2290.582	*Min* = -2402.135 *Max* = -2290.582	*Min* = -2417.993 *Max* = -2272.490	*Min* = -2417.992 *Max* = -2272.490	*Min* = -2417.992 *Max* = -2272.490
Number of free parameters	8	9	11	8	9	11
p-values	NA	vs. one ratio: *Min* = 1.54e-24 *Max* = 1	vs. one ratio: *Min* = 1.5e-40 *Max* = 1 vs. two ratio: *Min* = 0.999 *Max* = 1	NA	vs. one ratio: *Min* = 0.973 *Max* = 1	vs. one ratio: *Min* = 0.790 *Max* = 1 vs. two ratio: *Min* = 0.593 *Max* = 1
ω range	0.0001 – 1.267	Asexual: 0.0001 – 2.313 Sexual: 0.0001 – 0.788	Sexual 1: 0.0001 – 1.332 Sexual 2: 0.0001 – 1.674 Asexual 1: 0.0001 – 2.219 Asexual 2: 0.0001 – 1.644	0.0001 – 3.677	Asexual: 0.0001 – 2.313 Sexual: 0.0001 – 0.845	Sexual 1: 0.0001 – 0.674 Sexual 2: 0.0001 – 0.918 Asexual 1: 0.0001 – 0.738 Asexual 2: 0.0001 – 1.549

Supplementary Table 8: Summary of *codeml* analyses and comparisons of ratios in single copy orthologs between the parental species (*Aspidoscelis marmoratus*, *A. septemvittatus*), the asexual subgenomes of *A. tesselatus*, and three nonparental sexual species (*A. gularis, A. arizonae, A. sexlineatus*).

**Model**	**ln(L)**	**Number of free parameters**	**p-values**	***ω***
One ratio	-37390.131	14	NA	0.38599
Two ratio (sexual vs. asexual)	-37334.753	15	vs. one ratio: 6.68e-26*	Asexual: 0.17989<br>Sexual: 0.47938
Three ratio (parental sexual vs asexual vs nonparental sexual)	-37334.603	16	vs. one ratio: 7.67e-25*<br>vs. two ratio: 0.584	Parental: 0.1749<br>Asexual: 0.4794<br>Nonparental sexual: 0.1971
Seven ratio (individuals)	-37328.688	20	vs. one ratio: 4.034e-24*<br>vs. two ratio: 0.033*<br>vs. three ratio: 0.0186*	*A.marmoratus*: 0.1742<br>*A.septemvittatus*: 0.1974<br>*A.tesselatus-marm*: 0.5152<br>*A.tesselatus-sept*: 0.3942<br>*A.gularis*: 0.0708<br>*A.arizonae*: 0.2485<br>*A.sexlineatus*: 0.1597

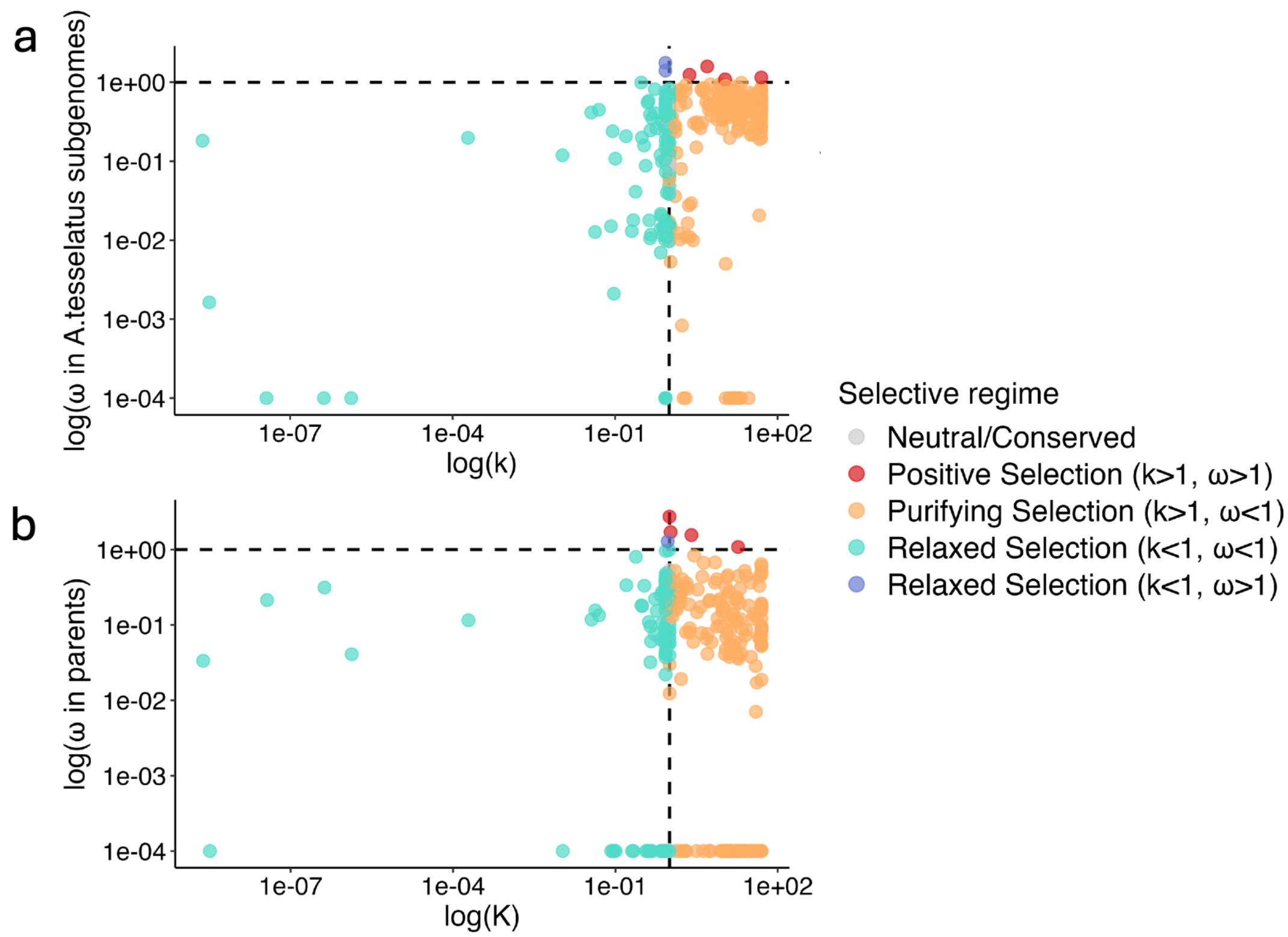

Supplementary Figure 1: Relationship between selection intensity (k) and branch-specific ω across orthologs. **a:** Relationship between k and ω in the *A. tesselatus* subgenomes. **b:** Relationship between k and ω in the parental branches. In both panels, the vertical dashed line indicates k=1, and the horizontal dashed line indicates ω=1. Points are colored by selective regime based on RELAX k and ω values. N=353 orthologs.

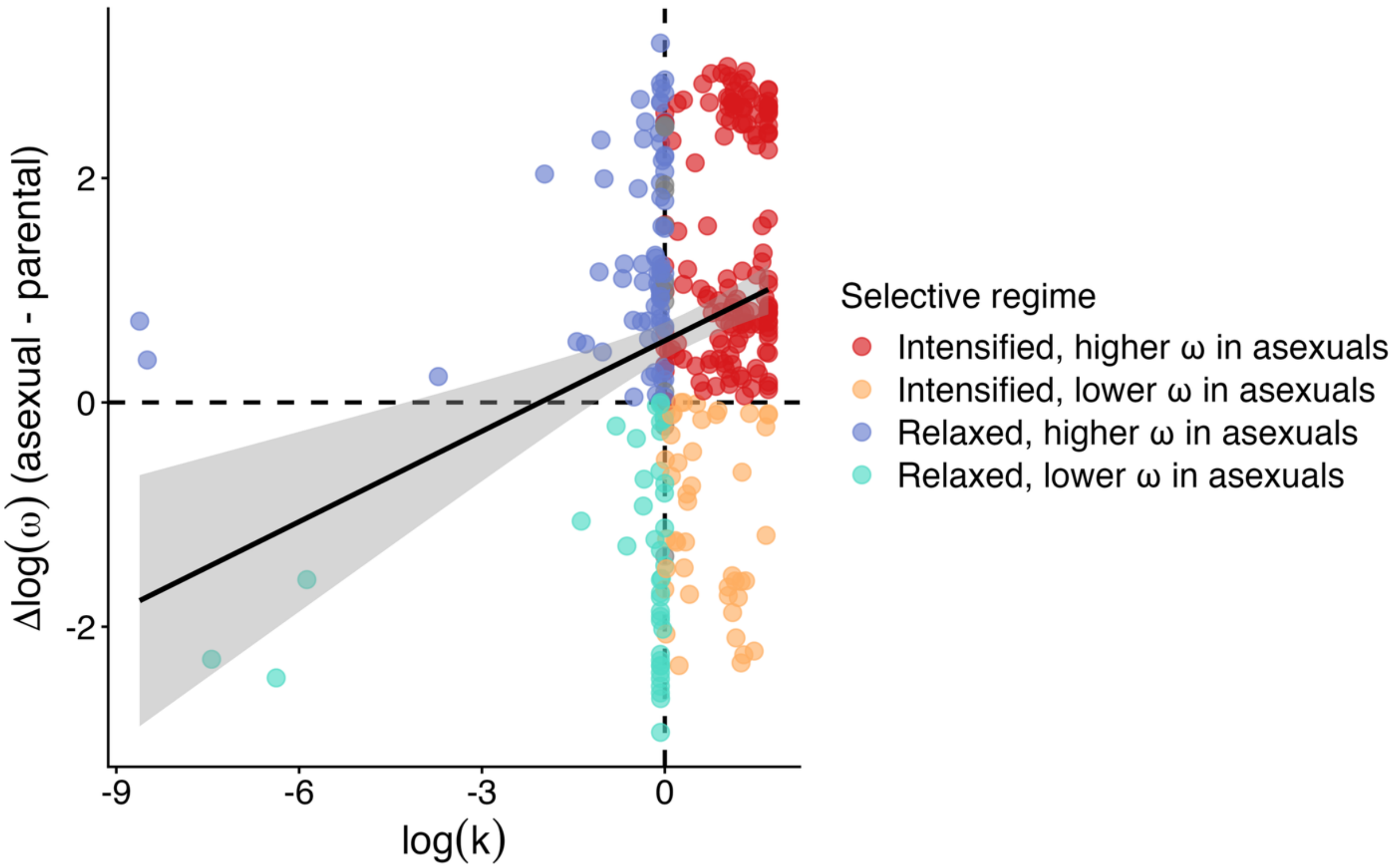

Supplementary Figure 2: Relationship between the RELAX selection intensity (k) and the change in ω between the asexual and parental branches ($\Delta \log(\omega) = \log(\omega_{asexual}) - \log(\omega_{sexual})$). The solid line represents the fitted linear model describing the relationship between k and the ($\Delta \log(\omega)$, with the shaded region indicating the 95% confidence interval. The horizontal dashed line indicates $\Delta \log(\omega) = 0$, separating reduced ω in the asexual branches compared to the sexuals ($\Delta \log(\omega) < 0$) from increased ω in the asexual branches ($\Delta \log(\omega) > 0$). The vertical dashed line indicates log(k) = 0, separating intensified (log(k) > 0) from relaxed (log(k) <0). Points are colored by inferred selective regime. (N = 353 orthologs).
